# Concept Mapping STI/HIV Prevention and Condom Use among Young African American Adults

**DOI:** 10.3390/bs14060501

**Published:** 2024-06-14

**Authors:** Chakema Carmack, Sarah Nganga, Eisha Ahmed, Taylor Coleman

**Affiliations:** 1 Psychological Health and Learning Sciences Department, University of Houston, Houston, TX 77204, USA; eishaahmed263@gmail.com (E.A.); tmcolema@cougarnet.uh.edu (T.C.); 2 Health Research Institute—Research Center in Minority Institution (HRI-RCMI), University of Houston, Houston, TX 77204, USA; 3 Center for Health Equity and Evaluation Research (CHEER), Texas A&M University, College Station, TX 77843, USA; 4 Legacy Community Health, Houston, TX 77009, USA; sarah.nganga@outlook.com

**Keywords:** STI risk, HIV prevention, African American, self-efficacy, condom use, concept mapping, social media

## Abstract

Theory-based HIV prevention programs have resulted in increased condom use, which remains the best method for the prevention of sexually transmitted infections (STIs) among sexually active heterosexual individuals. Particularly, the integrative model of behavior prediction theorizes that attitudes, norms, self-efficacy, and socioenvironmental factors influence intention and behavior and has been useful in understanding STI risk among adolescents. However, more research is needed regarding young African American adults. Given the increased freedom and decision-making independence afforded to young adults compared to adolescents, it is important to consider the STI/human immunodeficiency virus (HIV) prevention messages that would resonate with them, particularly regarding condom use. The present study sought to explore how attitudes, subjective norms, self-efficacies, and socioenvironmental factors may influence condom use and STI/HIV prevention, as conceptualized by the participants. We conducted a group-based concept map, a systems-thinking mixed methodology that resulted in a geospatial map reflecting the conceptualizations of the participants. Self-identified young heterosexual African American adults (*N* = 43) aged 20–26 engaged in an interactive concept mapping procedure in order to “map out” their overarching concepts about STI/HIV risk and condom use. Seven overall conceptual domains emerged: self-efficacy for partner communication, condom use self-efficacy, social media/sociocultural influences, condom use/STI knowledge, condom use cons, condom use pros, and subjective and social norms about condom use. We presented the concept map and discussed the conceptual interpretations and the relationships among the overarching concepts. We also discussed how the social environment, including the social media environment, was conceptualized regarding STI/HIV risk and prevention among young African American adults. Concept mapping can be viewed as a way to determine worthwhile messages for intervention development. The findings may provide information for prevention programs aimed at reducing the incidence of STIs among young adult heterosexual persons within African American communities.

## 1. Introduction

In the U.S., African Americans suffer higher rates of human immunodeficiency virus (HIV) incidence and comprise a higher proportion of persons living with HIV than their racial/ethnic counterparts [1]. African Americans make up about 13.6% of the population [2], but make up over 42% of HIV cases [3]. African Americans are almost eight times more likely to be diagnosed with HIV than their White counterparts [3]. Disparities can be found in gender/race comparisons also. African American men are eight and six times more likely to be living with HIV and die from HIV, respectively, than their White counterparts, while African American women are fifteen times more likely to be both living with HIV and die from HIV than their White counterparts [4]. Furthermore, African Americans are nine times more likely to have acquired immunodeficiency syndrome (AIDS), which is caused by HIV [5]. In 2018, heterosexual sexual contact comprised 32% of new cases among African Americans [5]. Thus, heterosexual African Americans are also in need of attention regarding prevention efforts.

Many barriers to health care exist, including HIV testing, poverty, lack of prevention knowledge, access to clinics, stigma, and lack of education [6]. Longstanding racism and discrimination also impact African Americans’ disease burden, including HIV prevention and care [7]. Many barriers can be conceptualized through understanding the social determinants of health, which are the conditions in which we are born, live, play, and work. Community environments psychologically shape our attitudes and behavior about prevention and health. Low-income, low-resource, and/or underserved communities are less likely to prioritize HIV prevention, and these areas often correlate with minority status in the U.S. [7]. Clearly, there is a need to understand how to engage African Americans in HIV prevention and testing that considers their unique minority experiences.

### 1.1. Evidence-Based HIV Interventions

Evidence-based and evidence-informed HIV interventions tailored to African Americans have found positive results in increasing HIV testing [8], condom use [9,10,11], PrEP use [12,13], and other prevention behaviors. However, out of the 31 CDC evidence-based and evidence-informed HIV interventions, the majority are tailored to men who have sex with men (MSM) and adolescents [14]. This is not surprising, considering that MSM make up the majority of African American HIV cases, and adolescence is an important developmental stage before or during the exploration of sexuality and sexual awareness. While targeting these high-risk groups is beneficial for addressing the HIV disparities among them, few evidence-based and evidence-informed interventions target young heterosexual African American adults. Young adulthood, ages 18–26 [9], constitutes a critical developmental period as well [15].

Many of the evidence-based and evidence-informed HIV prevention interventions utilize a variety of intervention methods and theory-based content. However, there are only a few that target heterosexual African American adults. Sister-to-Sister [16], an evidence-based HIV risk reduction intervention, applied the social cognitive theory (SCT) to one-on-one and group skill-building and STI/HIV knowledge among African American women. Sister-to-Sister utilized SCT constructs such as self-efficacy, collective efficacy, and agency as central features of prevention behaviors [17]. The intervention found success in increased condom use and reduced unprotected sex and new STD infections among the intervention groups. Focus on the Future is an evidence-based intervention that has been successful in showing greater condom use and fewer sexual partners among African American men assigned to the intervention [9]. The intervention reported fewer sexual partners, and participants were less likely to acquire an STI at follow-up [9]. Focus on the Future uses the information, motivation, and behavioral skills (IMB) theory, which posits that treatment effects on behavior occur largely as the result of treatment effects on behavioral skills, which follow from effects on information and motivation [17]. Eban [18], another evidence-based HIV prevention intervention, utilized the SCT in an Afrocentric paradigm and nested ecological theory to demonstrate self-efficacy and model goal setting and role play for heterosexual African American adults.

### 1.2. Theory and Intervention Messaging

Many useful psychosocial theories exist that may be used to address HIV prevention interventions. Self-efficacy and attitudes, for example, are prominent constructs found in many popular behavior theories useful for HIV prevention messaging, though more theoretical underpinnings based on environmental constraints are needed. The integrated model of behavioral prediction (IM) is a theory of behavior that integrates determinants of health behavior from various empirically tested behavioral theories, including the social cognitive model, health belief model, and theory of reasoned action [19,20]. The IM integrates aspects of intrapersonal beliefs (attitudes), social cognition (social and subjective norms), and self-efficacy. Also, the IM is preferred because it considers skills and environmental constraints that influence intention and behavior. As a foundation for intervention messaging, the IM is particularly advantageous for minority populations (e.g., African Americans) for whom certain social determinants of health will likely influence the skills needed to carry out the behavior (e.g., education, sexual literacy) and environmental conditions that may constrain the behavior (e.g., testing clinic access).

The IM states that intention is the proximal determinant of behavior. Intention is influenced directly by three constructs: attitudes, subjective norms, and self-efficacy. Attitudes about health behaviors are valuations on whether the behavior is perceived positively or negatively, whether the behavior is perceived as beneficial at a particular moment, and outcome expectations. Subjective norms are the degree to which one perceives that others important to them would want them to carry out the behavior. Subjective norms encompass motivations to comply, as well as the perception that these “important others” actually carry out the particular behavior. Self-efficacy is known as the perceived situation-specific ability to carry out a behavior and includes perceived behavioral control. The weight or importance placed on the proximal determinants of intention (attitude, subjective norms, and self-efficacy) determines the strength of the intention. Although intention is the proximal determinant of behavior, skills and environmental resources must coalesce for behavior to manifest from a strong intention.

The IM takes behavior prediction a step further by specifying the addition of skills and environment in its ability to predict actual behavior. Theoretically, intention should always predict behavior. However, when intention fails to predict behavior, the model is not in question and remains suitable as long as the antecedents remain substantial in predicting intention. The model also considers that skills and environment play a moderating effect on intention ultimately leading to behavior. Lack of sufficient skill and/or barriers within the environment may impede the carrying out of a particular behavior even if intentions may be strong.

The IM has found utility in intervention message development among African American adolescents [21,22]. Utilizing the antecedents of behavior (attitudes, subjective norms, self-efficacy, and intention) has shown significant decreases in STI risk behaviors and increases in protective behaviors, such as increased condom use, greater self-efficacy, decreased sexual activity, less anal sex, pro-condom attitudes, and greater HIV knowledge [21,22,23,24]. Sexually active African American adolescents who engaged in a safer sex education program utilizing the IM antecedents showed significant decreases in sexual activity at three-month follow-up compared to the abstinence-only group and greater condom use than the control group in a three-arm randomized controlled intervention trial [22]. Meanwhile, attitudes and subjective norms have shown greater impacts on intention among male African American adolescents, while subjective norms and self-efficacy have shown greater impacts on intention for female African American adolescents [21]. Nevertheless, the overall IM model can be used with confidence when applied to African American adolescents’ condom use intentions and behavior [21].

It would indeed be ideal for young people to understand STI risk and prevention prior to becoming an adult when there is newfound freedom for individual decision-making and less parental oversight. Although the World Health Organization recognizes age 19 as the end of adolescence, some researchers contend that adolescence, from a cognitive developmental sense, extends well up to 24 years old [25,26,27]. Notwithstanding, more research is needed regarding sexual risk behavior prevention among young African American adults, as their maturity, life experience, and cognition might still reflect the propensity for risk-taking akin to that of late adolescence.

### 1.3. Group Concept Mapping

One of the most difficult aspects of intervention development is often planning exactly what the intervention will focus on and teach or demonstrate [28]. Intervention programs are planned for a finite amount of time with a finite amount of resources. Therefore, the initial conceptualization of the intervention must be solid. For example, there may be little value in conceptualizing an abstinence program for at-risk youth who are already engaged in sexual activity to address STI disparities. Thus, exactly what the intervention will focus on should be tailored to the needs of the population of interest and ideally meet participants where they are in terms of their behavior and the social determinants of health that affect them. The benefits of group concept mapping are that it is a reliable method for participatory research, the results are immediately usable, and they reflect the values and nuanced contexts of the priority population regarding the issue being addressed.

Group concept mapping is a systems-thinking mixed-methodology procedure that utilizes “structured conceptualization” [28] to yield a graphical illustration of a particular focus topic that is conceptualized directly by the participants. The process is interactive in that participants discuss and develop a list of concepts related to an overall focus prompt (example focus prompt: What are some things about condoms that make you want to use them?). This list of concepts is reviewed by the researcher(s) *alongside* the participants. Thus, the participants’ unique values and context related to the topic are captured within the resulting model. Upon developing a list of pertinent concepts, participants are asked to sort and rate them. The entire group concept mapping procedure, from participant interaction to analyses and final map development, is carried out through a series of steps that are further discussed in the Methods section. Concept mapping results in a graphic model that is conceptualized by the target group themselves and is useful for illustrating the interconnectedness of how a set of constructs relate to one another (i.e., the “system”) [28,29,30].

This provides benefits beyond simple survey results. Quantitative survey methodologies may provide empirical interpretations and relative estimates of behavior among populations (e.g., factor analyses). However, it cannot illustrate the interconnectedness of the behavioral constructs, nor can it illustrate the size and scope of the constructs according to the priority population with the ease of use and ease of interpretability that group concept mapping offers. Most importantly, quantitative survey methods do not typically value the unique context of a particular population unless they have been created, normed, and validated specifically for that particular population. This poses a disadvantage to researchers whose work may include hard-to-reach or special populations. Although group concept mapping may limit broad generalizability, it is immediately useful for its planning (or evaluation) purposes, in that the results are inherently tailored to the priority population.

### 1.4. Study Purpose

As young adulthood remains a period of rapid change socially and developmentally, it is worthwhile to continue prevention efforts throughout this period. STI/HIV prevention interventions that include messaging specifically tailored to the intended audience may find greater utility in achieving their desired outcomes [21]. The present study utilizes concept mapping, an adaptive systems-thinking mixed methodology, to understand how young heterosexual African American adults conceptualize the IM determinants as they relate to STI/HIV prevention and condom use. Concept mapping is advantageous because it allows us to understand their beliefs about STI/HIV prevention and how the IM determinants operate in the minds of the participants, allows for the identification of new information, and is useful for program planning and intervention messaging [28,31].

The objective of the present study was to (1) show proof of concept for the utility of group concept mapping among young heterosexual African American adults, and (2) identify pertinent areas of intervention focus on condom use and HIV prevention in Black communities.

## 2. Materials and Methods

### 2.1. Participants

The study sample included 43 young African American adults aged 20–26. The mean age was 22.4, with a standard deviation of 1.4. All participants (*N* = 43) reported being of non-Hispanic African American race/ethnicity. Twelve percent (*N* = 5) of the sample reported having a bachelor’s degree, 51% (*N* = 22) of the sample reported having some college education/being currently in college; 30% (*N* = 13); and 7% (*N* = 3) reported having a high school diploma. Half of the sample (50.2%, *N* = 22) reported having private insurance, and 49.8% (*N* = 21) reported having no insurance.

### 2.2. Procedure

The study was approved by the university’s internal review board. Eligibility included self-reported heterosexual African American aged 18 to 26 years and not previously diagnosed with an STI. Participants were recruited through university websites, community center flyers, digital postings, and word of mouth. Interested and eligible participants were contacted via email about participation. Eligible participants were informed that study participation would occur at a well-known local community center located within one of our priority recruitment areas. The research team carried out recruitment efforts for approximately two months with the support of five local community organizations with positive long-standing relationships in our priority communities. Through these recruitment efforts, we tentatively scheduled 48 participants for participation on the predetermined study facilitation day. In total, 33 of the 48 tentatively scheduled participants showed up to participate on the study facilitation day. Study participation occurred (purposefully) during an unrelated community event. Thus, the research team was able to recruit 12 additional participants on-site during the community event. Participation was carried out over two separate sessions that entailed unique phases of the concept mapping procedure: (1) the information-gathering session (phase 1) and (2) the sorting–rating session (phases 2 and 3). Overall, the study included 45 participants who participated in phase 1 and 43 participants who participated in phase 2 (two participants in phase 1 of the procedure could not be scheduled for phase 2).

Participants gathered in a private conference room at a local community center. Participants were given the consent form and a research assistant read the consent aloud for participants. Participants were asked if they had any questions about the study’s aims and/or procedures. Upon providing written consent to participate, participants began the first session. Session 1 entailed that participants engage in an open conversation, given study-related prompts, to gather pertinent “ideas” about the subject matter. A trained facilitator moderated the session. The phase 1 discussion was carried out in a closed-door conference room with all participants together, both male and female. The trained facilitator has over ten years of experience in qualitative group discussion methodologies (e.g., focus groups, group-based interviewing, etc.). The facilitator introduced herself and the study and ensured participants that their discussion points would remain confidential. The facilitator reminded participants to be mindful and respectful of divergent opinions and to refrain from sharing the discussion beyond the study session, given the topic of discussion. Participants were also informed that they did not have to engage in any prompt or discussion with which they did not feel comfortable. Participants were asked to identify themselves before speaking with an alias to increase confidentiality. Four research assistants assisted in the study facilitation by taking notes of particularly highlighted conversation topics and other notes (e.g., perceptions of majority agreement via head nods, for example) commonly taken during qualitative group methodologies (e.g., focus groups). The trained facilitator made concerted efforts to ensure that all participants had a voice in the discussion, as they chose, with non-verbal techniques to increase group engagement (e.g., walking around the room, speaking very conversationally, eye contact, etc.). Participants were routinely asked if there were any other considerations regarding a particular prompt before moving on to the next. Notes taken by the research assistants noted specific comments from at least 29 of the 43 participants, although there were likely more participants actively conversing throughout who were not identified specifically through research assistant notes.

For this open conversation, the following prompts were used: How do you feel about using condoms during sexual activity?; What are some things that would make you want to use a condom?; What are some things that make you not want to use a condom?; What would make it easier to use condoms?; and How detrimental are STIs to you and/or the community? These prompts were written on a whiteboard to organize the ideas that emerged. Each prompt was discussed for approximately 15 min. The entire session 1 took approximately 1.5 h. During the session 1 discussion, short phrases that reflected the ideas related to the prompts were recorded by a dedicated research assistant on an interactive whiteboard. After collecting all pertinent phrases, the research assistants prepped for session 2 with the participants. This entailed electronically transcribing the phrases onto individual cards. Predetermined phrases with strong associations with condom use intention that reflected antecedents of intention according to the IM theory were also electronically transcribed onto individual cards prior to study facilitation. The resulting set of cards resulted in 50 phrases generated from session 1 and 17 predetermined phrases reflecting the IM theory that had not emerged during session 1 discussions, for a total of 67 individual concept cards. Each phrase on the card sets was numbered for identification. Ten card piles were created, which took the research team approximately 30 min to prepare on-site.

At the end of phase 1 participation, participants were scheduled ten at a time, in one-hour increments, to complete participation in session 2 (phases 2 and 3) later that day or at another time convenient to them. Of the 45 participants who participated in phase 1, 31 of them returned throughout the day to complete phase 2 and 11 participants were scheduled to complete their individual ratings at a later date, which they did.

Phase 2 of the concept mapping procedure entailed sorting the concept cards into piles. Sorting rules were (a) there is no limit to the number of piles that could be created, and (b) there must be more than one card per pile. Phase 3 entailed naming and rating each pile. Participants were then given a sorting–rating sheet to record the card numbers for each pile they created and to provide a title for each pile. Basic demographic information was included at the top of the sorting–rating sheet. They were then instructed to rate the importance of each concept in an HIV prevention education program. This phase took approximately 40 min. Upon completion, participants were thanked for their participation and were told to help themselves to light refreshments provided by the research team. Although no compensation was provided, participants were given a “swag bag” provided by our community partners collectively that included various promotional items from our community partners: a local community organization T-shirt, water bottle, notepad, and keychain.

### 2.3. Measures

Card phrases and rating–sorting sheets served as the measures for the current study. Predetermined card phrases identifying attitudes, subjective norms, and self-efficacy regarding condom use (integrative model) that were not mentioned in the session 1 discussions were garnered from previous studies that have shown strong associations with condom use intention [21,22,23]. Table 1 shows the 67 phrases that emerged from session 1 discussions and predetermined phrases that were adopted from previous research. The sorting sheet contained 10 blank boxes for 10 possible piles. Participants were instructed to ask for another sorting sheet if they intended to make more than 10 piles. Instructions printed on the sorting sheet were to record the card numbers for each pile in its own individual box, then come up with a name for the pile, with one pile of cards per blank box. The rating sheet listed each phrase set on a Likert-like scale of 1 (not very important) to 5 (very important). The instruction printed on the rating sheet was “Please rate how important each concept would be in addressing STI/HIV prevention.” Age, gender, education level, and insurance demographics were also collected on the rating sheet.

### 2.4. Analytic Procedure

Phases 4–6 of the concept mapping procedure entailed the analytic procedure. The concept map was analyzed and illustrated using Concept Systems software, an academic/professional software package specifically designed to analyze the concept mapping procedure and data. Phase 4 of the concept mapping procedure entailed creating a similarity matrix to identify conceptual domains. Phase 5 entailed the use of multidimensional scaling to create point maps and cluster maps. At this point, the research team examined various cluster maps, starting with 10 clusters [28]. Upon examination, seven clusters were agreed upon and named based on the individual concepts that loaded under each cluster. This process (phase 5) is akin to qualitative thematic analysis, in that the configuration of clusters is chosen based on the conceptualization that “makes the most sense,” in a similar way to qualitative themes being named. Phase 6 arranged the average participant ratings for each statement and was graphically overlain onto the clusters. This yielded a final concept map.

## 3. Results

**Figure 1:**
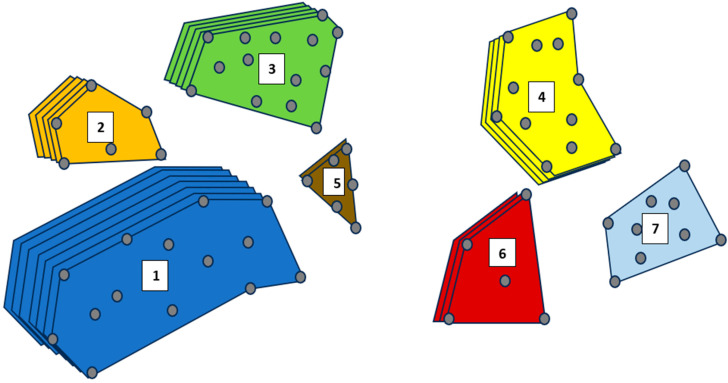
Final concept map. 1. Self-efficacy for partner communication. 2. Condom use self-efficacy. 3. Sociocultural/social media influences. 4. Condom use/STI knowledge. 5. Condom use cons. 6. Condom use pros. 7. Subjective and social norms about condom use.

The final concept map yielded a set of concepts graphically illustrated (Figure 1), known as conceptual domains. The shapes represent the different conceptual domains about condom use, as they are conceptualized by the participants aggregately. Shapes with more layers indicate that there was high agreement on the importance of the conceptual domain. Fewer layers indicate that the group did not rate the conceptual domain as important in comparison with the other conceptual domains. Conceptual domains that are more “spread out” geometrically indicate that the individual phrases within domain are more spread out in the minds of the participants. Smaller shapes indicate that the individual phrases are more succinct in the minds of the participants. The location of each conceptual domain provides information about its relative association with other conceptual domains. Thus, conceptual domains that are closer together are more related to one another, and those that are far apart are less related to one another in the minds of the participants. Lastly, the nodes (i.e., dots) are the individual phrases/statements that were sorted and rated. The relative position of the nodes indicated their relative association with one another within a conceptual domain as well as with all other individual concepts within the map.

Upon examining different cluster solutions, we determined that a seven-cluster solution would be preferable. We examined whether there were demographic differences regarding the clusters. No significant mean differences were found between gender, insurance status, or education level in any of the clusters. The resulting cluster map (Figure 1) showed how these young adult African American men and women conceptualized condom use and STI/HIV prevention. Upon selecting a cluster solution, the conceptual domains were named and ranked. As such, a hierarchy of the relative importance of each conceptual domain can be observed in Figure 1: (1) self-efficacy for partner communication; (2) condom use self-efficacy; (3) sociocultural/social media influences; (4) condom use/STI knowledge; (5) condom use cons; (6) condom use pros; and (7) subjective and social norms about condom use. Table 1 shows the conceptual domains, the individual concepts that loaded within each, an average importance rating of each conceptual domain, and an average importance rating of the individual concept. Items were averaged on a 5-point Likert-like scale: closer to 1 indicated that the individual concept was not very important, and ratings closer to 5 indicated that the concept was very important. Below, the bracketed values indicate the average importance rating for the individual concepts they follow.

The “Self-efficacy for partner communication” conceptual domain was rated the most important conceptual domain and resulted in an average importance rating of 4.31. The most important individual concept of STI/HIV prevention was “Talking to partner about condoms before sex” [4.8]. The second-equal most important concepts within this domain were “Using a condom without ruining the mood” [4.7] and “Refusing sex if a guy or girl has no condom” [4.7]. The least important concept within this domain was “Trusting partner not to have sex with other people” [2.7]. The “Condom use self-efficacy” conceptual domain was the second-most important conceptual domain and resulted in an average importance rating of 3.80. The most important individual concepts within this domain were “Emotional maturity needed before engaging in sex” [4.8] and “Being proud to practice safe sex” [4.8]. The least important concept in this domain was “Can get a condom from friends” [2.2]. The “Sociocultural/social media influences” conceptual domain was the third-most important and resulted in an average importance rating of 3.70. The most important individual concept for STI/HIV prevention within this conceptual domain was “HIV and testing discussed in sex ed/PE/health class [4.8] and HIV a problem for the Black community” [4.8]. The second-most important individual concept was “STI testing should be promoted on social media” [4.6] and “’Social media ‘STI/HIV Testing Challenge’” [4.6] (elaborated upon in the Discussion below). The least important concept was “Couples getting tested together” [2.0]. The “Condom use/STI knowledge” conceptual domain resulted in an average importance rating of 3.54. The most important concepts within this domain were “Black people die from AIDS more than other racial groups in the U.S.” [4.8] and “Knowing your status means you get medical care sooner rather than later” [4.7], while the least important concept was “PrEP prevents STIs” [1.0].

The “Condom use cons” conceptual domain resulted in an average importance rating of 2.91, and the “Condom use pros” conceptual domain resulted in an average importance rating of 2.65. The most important concept regarding condom use cons was “Condoms cost too much” [4.6], while the least important concept was “Thinking that a woman carrying condoms means she is promiscuous” [1.0]. The most important pro-condom concept was “Cost of STI/HIV medication” [4.5], while the least important pro-condom concept was “Women will think a man is responsible if he carries condoms” [1.5]. The “Subjective and social norms about condom use” conceptual domain was the least important overarching concept regarding STI/HIV prevention and resulted in an average importance rating of 2.29. The most important concept within the “Subjective and social norms” conceptual domain was “Would consider PrEP if I had a friend already taking it” [3.6], while the least important concept was “My parents wanting me to use a condom” [1.2].

## 4. Discussion

Concept mapping was used in the present study to examine psychosocial, socioenvironmental, and knowledge concepts about STI/HIV prevention and condom use among young adult African American adults. Concept mapping results in the conceptualization and organization of a particular topic directly developed from the minds of the participants. In the present study, the final concept map resulted in seven conceptual domains: self-efficacy for partner communication, condom use self-efficacy, social media/sociocultural influences, STI/HIV and condom use knowledge, condom use cons, condom use pros, and subjective and social norms about condom use. Phrases generated in phase 1 (session 1), in which the conceptual domains were composed, reflected the beliefs about the participants’ attitudes, subjective norms, and efficacies about condoms, as well as beliefs and opinions about traditional gender roles, relationship dynamics, STI knowledge, PrEP, and social media. Concept mapping has been demonstrated to be useful for planning purposes [28] and initiating community program development, and has been used as a method for engaging patients in primary care practice improvement [28,29,31,32]. However, it was unclear if this methodology is feasible for obtaining useful and/or new information about STI/HIV prevention among heterosexual African Americans. Results of the present study, along with the feasibility of carrying out the group procedure, showed proof of concept in obtaining both useful and new information about STI/HIV prevention among this group. We further elaborate on the congruence with theory, new information attained, and how these results may be immediately useful for community intervention programs below.

The majority of the phrases generated were congruent with the IM’s determinants of intention and behavior moderators (skills and environment). The “Self-efficacy for partner communication” conceptual domain emerged as the highest-rated overall concept (4.31/5) as it relates to its importance in STI/HIV prevention intervention and education. These concepts involved behaviors that would be needed to communicate condom use desires and requirements to young adults’ sexual partners. Particularly, the concepts rated with the highest importance had to do with talking to partners about condoms *before* sex, being able to use condoms without ruining the mood, stopping physical intimacy to put on a condom, and getting a partner to agree to condom use. However, not all the concepts that emerged under this conceptual domain involved condom use specifically. Fear of disclosing a positive STI status, talking about condoms as evidence of caring about oneself, and condom use bringing couples closer also emerged within this conceptual domain. As the most important conceptual domain created by the participants, this is in line with previous studies that examined the importance of partner communication for minimizing sexual risk. Previous research concluded that low self-efficacy and lack of conversations about condoms before sexual encounters resulted in decreased condom utilization [23,33], whereas frequent discussions about condoms predicted more frequent condom use [34]. Likewise, having the self-efficacy to communicate about condom use was associated with more positive attitudes about condoms [35], and STI disclosure was found to be greater among those who had greater emotional ties to their partners [36]. Interestingly, the participants did not think it was important to discuss their partner’s trust regarding monogamy, although this concept emerged within this conceptual domain in all possible map configurations.

As it relates to the IM of behavioral prediction theory [19], the subjective norms and self-efficacy constructs emerged largely within their own conceptual domain in the minds of the participants. However, the attitudes construct, as in positive or negative attitudes about condom use, was not heavily discussed in the phase 1 information-gathering phase. Many of the concepts indicating attitude that were rated by the participants were predetermined concepts from previous studies on condom use behavior and IM constructs. Attitudes about condoms and STIs are thought to play a pivotal role in condom use behavior, as previous research found that individuals with positive perceptions of condom use had utilized condoms during their most recent sexual encounters [37]. One specific concept of attitude, “Partner not liking to use condoms,” was rated highly important (4.7/5.0), but was subsumed within the “Self-efficacy for partner communication” conceptual domain. Interestingly, the essence of the concept centers on the partner’s attitude about condom use, not one’s own attitude about condoms. A few concepts touched on the benefits of condom use (e.g., “Condoms can prevent HIV”), which is indicated in the attitudes construct according to the IM, but were subsumed within other conceptual domains by the participants here. “Subjective and social norms about condom use” was the least important conceptual domain. It is interesting that the participants rated “My parents would want me to use a condom” as the least important concept in this conceptual domain, as well as one of the least important concepts within the entire map. This could further reflect the independence and identity formation that continues to develop during this period.

Skills and environmental constraints, which are moderators of behavior in the IM, were mostly subsumed within the “Condom use self-efficacy” conceptual domain. These concepts included having the resources and money to physically acquire condoms, carrying a condom, the proper use of a condom, and recognizing the maturity needed in positive sexual relationships. Upon visual inspection of the concept map, the ”Condom use self-efficacy” conceptual domain was closest to the “Self-efficacy for partner communication” conceptual domain, meaning they are highly related concepts in the minds of the participants, but certainly separate. This is in line with previous research that found that having the self-efficacy to use condoms may increase partner communication about condom use desires/requirements and STI disclosure [36].

Sociocultural and social media factors emerged as a conceptual domain not specifically captured within the IM, constituting new information about how condom use is conceptualized among young heterosexual African American adults. Within the “Sociocultural/social media influences” conceptual domain, many behaviors and tools that could increase STI/HIV prevention behavior (i.e., HIV testing, PrEP knowledge and uptake, and condom use) could be observed. Within the “Sociocultural/social media influences” conceptual domain, the individual “Social media ‘STI/HIV Testing Challenge’” concept was discussed in phase 1 of the concept mapping procedure as a challenge involving being proud to talk about the importance of knowing your status on social media. This may be a way to cultivate a public health presence in social media spaces that may have a social networking effect and expanded reach. It is interesting that education about PrEP was included within this conceptual domain. This congruence is in line with Kudrati and colleagues, who found that social media platforms can serve as a promising approach to generating more PrEP awareness and participant engagement [38]. The advantages include affordability, accessibility, and usability, given the widespread use of social media. There is leverage in the flexibility that online platforms such as Instagram and Meta afford community engagement (e.g., community chat or “edutaining” online quizzes). Additionally, messaging and online dissemination regarding PrEP awareness are positioned to address misperceptions and concerns related to PrEP use through social media outlets [38].

Taken altogether, there were six individual concepts that were rated to be the most important (4.8/5.0) concepts within the map. Two of these concepts concerned HIV in the African American community (“HIV is an issue in the Black community,” and “Black people die from AIDS more than other racial groups in the U.S.”), and three concepts concerned general esteem for oneself (“Talking to partner about condoms means you care about yourself,” “Being proud to practice safe sex with condoms,” and “Emotional maturity needed before engaging in sex”). Intervention messaging for the current population would want to capitalize on and expound upon these concepts in developing intervention materials. Upon further visual inspection of the final concept map (Figure 1), a subtle distinction can be made within the matrix space. In other words, there was a distinction between conceptual domains clustered together on the left side of the figure and a group of conceptual domains clustered together on the right side of the figure. The conceptual domains of partner communication self-efficacy, condom use self-efficacy, sociocultural and social media influences, and addressing condom use cons (which contained many of the predetermined attitude concepts) were more related to one another than the others that seemed to group on the right. On the right side of the concept map (Figure 1), the conceptual domains of condom use/STI knowledge, condom use pros, and subjective and social norms about condom use seemed to cluster together in the minds of the participants.

### 4.1. Public Health and Practice Implications

The visual observations of the final concept map (Figure 1), discussed above, offer insights into practice implications by implicating particular topics of interest and concepts that may be leveraged to increase condom use and thereby decrease the incidence of STIs and HIV. Risk reduction interventions are conducted with finite resources. Understanding which topics to capitalize upon that are relevant to the intended audience is paramount to its success. According to the present study, spending a considerable amount of time on developing the skills for young people to communicate about the use of condoms before sex should be poignantly taught, demonstrated, and practiced within any STI risk reduction program for young African American adults. Integrating some sort of social media component within the intervention may also be useful to the success of the intervention. The present study suggests that young African American adults may be more engaged in interventions that incorporate elements of social media that discuss condom use in relationship dynamics, education about prevention (e.g., PrEP), and perceived norms related to condoms and condom use.

Young adulthood is a pivotal time to reinforce, change, and establish positive pro-condom behaviors. STI/HIV risk reduction is particularly important at this time, as this developmental period may typically include an increased number(s) of sexual partners, alcohol and drug experimentation, and unpredictable sexual situations [37,39]. Most sexually active young adults are not obtaining regular STI screening, nor are they counseled for healthy sexual practices. Moreover, risky behavior practices have been associated with the occurrence of STIs, HIV, and unplanned pregnancies. As a result, untreated STIs can lead to serious complications such as infertility, cancer, and an increased risk of organ damage. Previous research highlights various barriers to accessing proper sexual health and STI preventative services in youth populations across the U.S. Notably, lack of quality health insurance, transportation, cost, missed opportunities during patient visits, concerns of confidentiality, and embarrassment or shame are often reported as major barriers to maintaining sexual health and wellness via STI screening [40,41].

These social determinants of health are highly predictive of HIV testing behavior [40]. Public health and mental health professionals in the field are in an excellent position to address these concerns when it comes to HIV testing and STI prevention (i.e., condom use). Interventions and group or individual counseling may find it useful to incorporate some of these concepts in practice to counterbalance the deleterious effects that the social determinants of health may have on young African American adults. For instance, affording condoms, a concept that emerged within the “Condom use self-efficacy” conceptual domain, was rated with moderately high importance (3.7/5.0) by the participants. Interventionists and counselors may inform participants and clients about local clinics, organizations, or community sites that provide free and low-cost condoms. “Condoms should not be in locked cases in drugstores,” a concept that emerged within the “Sociocultural/social media influences” conceptual domain, was also rated relatively highly by the participants (4.1./5.0). Interventionists and counselors may guide participants and clients toward empowerment and self-efficacy in purchasing condoms and being “proud” of it (which was also rated as highly important (4.8/5.0) by the participants in this study), despite this physical and social normative barrier of condoms in locked cases. As it pertains to HIV testing, public health professionals have responded to the high STI rates in young people by attempting to develop accessible alternatives to traditional sexual health screening practices with self-test STI kits [38]. More research is needed on the efficacy, cost-effectiveness, and public promotion of STI/HIV self-test kits, as well as PrEP uptake, in which multimedia campaigns have been used to increase awareness [42].

The beauty of group concept mapping is that the final map directly shows what is important to the group, how important it is to them, what concepts they tend to cluster together in their mind, and how each concept is related to every other concept that they themselves conceptualized regarding the topic. As the results of group concept mapping may be immediately usable for intervention development, the present study identified priorities and recommendations for intervention within this population and context. Firstly, we recommend that partner communication about condoms and STIs should be a top priority for interventions tailored to this population. Partner communication may be a component in many STI risk reduction programs, but our results highlight that it should be central and foremost to the intervention material. Many interventions focus on condom use, which is observed here in the “Condom use self-efficacy” domain. However, here we see that those concepts (the self-efficacy to use a condom and the self-efficacy to talk to your partner about using a condom) are two separate concepts that may need concerted attention separately.

Secondly, we recommend the use of social media. Risk reduction programs will benefit from the use of social media creatively embedded within their program materials. Respectfully engaging in social media discourse about safe sex and encouraging risk reduction on social media is important to this young adult population. It has the potential to increase buy-in of the program while reducing risk behavior. Education and edutainment modules could be developed and implemented via social media platforms, creating a virtual community regarding safer sex and a safe space for discussion. Notwithstanding, from a public health perspective, social media may have far reach in its potential to influence health behavior. Thirdly, STI reduction should include discussions on PrEP—what it is, who it is for, and why it is important to consider—as wanting more education on PrEP was rated highly important as an individual concept within socioculture. This is especially important for young heterosexual African American adults who may erroneously believe it is only for homosexual persons. Our finding recommendation is discussing this information in a creative interactive way via social media, as these concepts concerning PrEP were closely related to STI/HIV awareness in social media spaces. Lastly, our results suggest that social norms should be less of a focus for condom use behaviors for young African American adults. This domain was rated the least important and was also least related, in concept, to condom use self-efficacy and self-efficacy for partner communication, which were the most important conceptual domains.

Additionally, various aspects of these domains can be further noted and used in intervention development. In teaching young African American adults about STI and HIV prevention, basic knowledge that many STIs can lie dormant in the body while still being transmitted through sexual intercourse is important to convey. It would be beneficial to explain STIs and HIV in the context of the Black community. Statements that were unique to STIs disproportionately affecting the African American community are highly important to young African American adults. Homing in on the emotional maturity needed for young adult sexual relationships would also be a worthwhile endeavor for prevention programming, as this was conceptualized by the participants as a point of discussion and rated highly.

### 4.2. Limitations

The following limitations of the present study are noted. Although there is no required sample size for this mixed-methodology procedure and analysis, a larger sample would bolster the results and interpretations. Concept mapping can be conducted with as few as 15 participants and as many as 60, although more than 60 may not be advised in typical settings to ensure that all participants feel invested in the session 1 group discussions [28]. Nevertheless, due to the qualitative and exploratory nature of the present study, these results should not be generalized without extensive replication and testing. Additionally, the results should be viewed or used with caution with other populations than presented in the sample here. There are various final maps that could have been developed from the present study data. The qualitative aspect of the present study allowed us to identify the number of clusters (i.e., conceptual domains) that made the most sense regarding how they grouped and the concepts captured therein. Thus, there are hypothetically other conceptualizations (i.e., final concept maps) that could have been observed.

## 5. Conclusions

Circumstances and behaviors in young adulthood have important implications for the transition to adulthood and the rest of one’s life. Attitudes and behaviors about health, economic security, and well-being during this period make it an excellent opportunity to solidify sexual health practices such as knowing your status and practicing safe sex with condom use. The present study used the integrated model of behavioral prediction (IM) applied to a concept mapping procedure on STI/HIV prevention and condom use beliefs. To date, there is a dearth of literature on the use of group concept mapping for behavioral program development regarding the African American STI/HIV disparity and significantly less attention overall regarding young heterosexual adults. This study is one of the first to address STI/HIV program planning with the use of group concept mapping specifically for intervention development among this priority group. The IM can offer interventionists and practitioners ways to maximize the correspondence between the intended population’s unique needs and the content of a message [19]. The visual–spatial observations of the final concept map along with the quantitative ratings presented here 1) hierarchically show which concepts to capitalize upon (via size, shape, and relative proximity) when developing content-effective STI risk-reduction interventions for this group and 2) offer insights into various testable hypotheses regarding their impact on actual condom use behavior following intervention implementation. Implementation of the findings will be explored through future research. Future research should also identify optimal social media platforms and approaches that would engender engagement and hold relevance, relatability, and public health accuracy for PrEP, HIV testing, HIV treatment and medication, and STI/HIV prevention through condom use behavior. Taken together, concept mapping methodology has been underutilized in risk-reduction intervention development, and the current study serves as a demonstration of its utility for tailored behavioral risk-reduction programming.

## Figures and Tables

**Table 1:** Conceptual domains (clusters), individual concepts, and importance ratings for the final concept map.

Conceptual Domain (Cluster) and Individual Concepts	Rating	Conceptual Domain (Cluster) and Individual Concepts	Rating
Self-Efficacy for Partner Communication	4.31	Sociocultural/Social Media Influences	3.70
Talking to partner about condoms before sex ^a^	4.8	STI testing should be on promoted on social media	4.6
Using a condom without ruining mood ^a^	4.7	Couples getting tested together a good thing	2.0
Can refuse sex if guy/girl has no condom ^a^	4.7	Social media “STI/HIV Testing Challenge”	4.6
Can get partner to use condom ^a^	4.6	Men carrying condoms does not mean promiscuous	2.3
Partner not liking to use condoms	4.6	My parents should have talked about me using condoms	4.0
Can stop sex to put on a condom/to ask partner to put on a condom ^a^	4.3	HIV is an issue in the Black community	4.8
Trusting partner not to have sex with other people	2.7	Condoms should not be in locked cases in drugstores	4.1
If partner does not want to use condom, you should question their STI/HIV status	3.5	Talking about sex with different partners on social media makes you more desirable	3.9 ^b^
Man having condoms means he is having sex with other people	3.7	HIV and testing discussed in sex ed/PE/health class	4.8
No use trying to use condoms with partner now if you didn’t start out using them	4.6	Having a “safe” confidant to talk to about sexual health issues	3.4
Varieties of condoms make them better to use	4.6	Sex is still taboo to older generations	2.3
Afraid to tell partners about current STI	4.7	More young adults should be on PrEP	3.0
Talking to partner about condoms means you care about yourself	4.8	More education about PrEP	4.2
Talking about safe sex with my partner brings us closer	3.9	Condom Use Self-Efficacy	3.80
Condom Use/STI Knowledge	3.54	Can afford condoms ^a^	3.7
Condoms can prevent HIV	3.6	Emotional maturity needed before engaging in sex	4.8
Besides HIV, other STIs are curable	2.6 ^b^	Bringing your own condom, no matter your gender, is always best	4.0
Sexually active young adults should get an HIV test	4.0	Being proud to practice safe sex with condoms	4.8
Plastic condoms are good if you are allergic to latex	3.0	Can get condom from a friend	2.2
Black people die from AIDS more than other racial groups in the U.S.	4.8	Properly putting on a condom/properly put a condom on my partner ^a^	3.3
Pregnancy more important than STIs to men	4.1 ^b^	Condom Use Cons	2.91
Oral sex and STIs	3.0	Condoms are embarrassing	3.2
Knowing your status means you get medical care sooner rather than later	4.7	Hard to carry condoms ^a^	2.5
Testing is important when you have multiple partners	4.0	Sex feels unnatural with condoms ^a^	2.9
Getting HIV does not mean you will die from it	4.0	Condoms breaking the rhythm of sex ^a^	3.1
HIV can go undetected in the body for years	4.6	Thinking that a woman carrying condoms means she is promiscuous ^a^	1.0
PrEP prevents HIV	2.6	Condom use means no trust in the relationship ^a^	2.7
PreP prevents STIs	1.0	Condoms cost too much ^a^	4.6
Subjective and Social Norms about Condom Use	2.29	Alcohol and drugs with sex makes condoms difficult	3.3
Having friends that use condoms	3.4	Condom Use Pros	2.65
Being a good person means disclosing a positive status to your partner	1.8	Having sex with a condom is better than not getting sex at all	2.1
Getting an HIV test would be easy for me to do	3.0	Cost of STI/HIV medication	4.5
Waiting to receive test results would only make me worry ^a^		Women will think a man is responsible if he carries condoms	1.5
My friends would want me to use a condom ^a^	2.0	“Hook-ups” more likely if I already have a condom	2.9
My partner would want us to use a condom ^a^	3.0		
My parents would want me to use a condom ^a^	1.2		
Would consider PrEP if I had friends already taking it	3.6		
Slut-shaming makes girls/women embarrassed about carrying condoms	2.7		

^a^ Phrases adapted from previous HIV prevention studies; ^b^ Item reverse coded for rating average.

## Data Availability

Data is publicly unavailable due to small sample size and privacy restrictions. Data requests may be submitted by contacting the corresponding author.
